# To the Land and Beyond: Crab Microbiomes as a Paradigm for the Evolution of Terrestrialization

**DOI:** 10.3389/fmicb.2020.575372

**Published:** 2020-10-07

**Authors:** Stefano Cannicci, Sara Fratini, Niccolò Meriggi, Giovanni Bacci, Alessio Iannucci, Alessio Mengoni, Duccio Cavalieri

**Affiliations:** ^1^Swire Institute of Marine Science and Division of Ecology and Biodiversity, The University of Hong Kong, Pokfulam, Hong Kong; ^2^Department of Biology, University of Florence, Florence, Italy

**Keywords:** brachyuran crabs, holobiont theory, symbiotic microbiota, comparative genomics, functional genomics

## Abstract

The transition to terrestrial environments by formerly aquatic species has occurred repeatedly in many animal phyla and lead to the vast diversity of extant terrestrial species. The differences between aquatic and terrestrial habitats are enormous and involved remarkable morphological and physiological changes. Convergent evolution of various traits is evident among phylogenetically distant taxa, but almost no information is available about the role of symbiotic microbiota in such transition. Here, we suggest that intertidal and terrestrial brachyuran crabs are a perfect model to study the evolutionary pathways and the ecological role of animal-microbiome symbioses, since their transition to land is happening right now, through a number of independent lineages. The microorganisms colonizing the gut of intertidal and terrestrial crabs are expected to play a major role to conquer the land, by reducing water losses and permitting the utilization of novel food sources. Indeed, it has been shown that the microbiomes hosted in the digestive system of terrestrial isopods has been critical to digest plant items, but nothing is known about the microbiomes present in the gut of truly terrestrial crabs. Other important physiological regulations that could be facilitated by microbiomes are nitrogen excretion and osmoregulation in the new environment. We also advocate for advances in comparative and functional genomics to uncover physiological aspects of these ongoing evolutionary processes. We think that the multidisciplinary study of microorganisms associated with terrestrial crabs will shed a completely new light on the biological and physiological processes involved in the sea-land transition.

## To the Land and Beyond: A True Crab Endeavor

**Figure 1:**
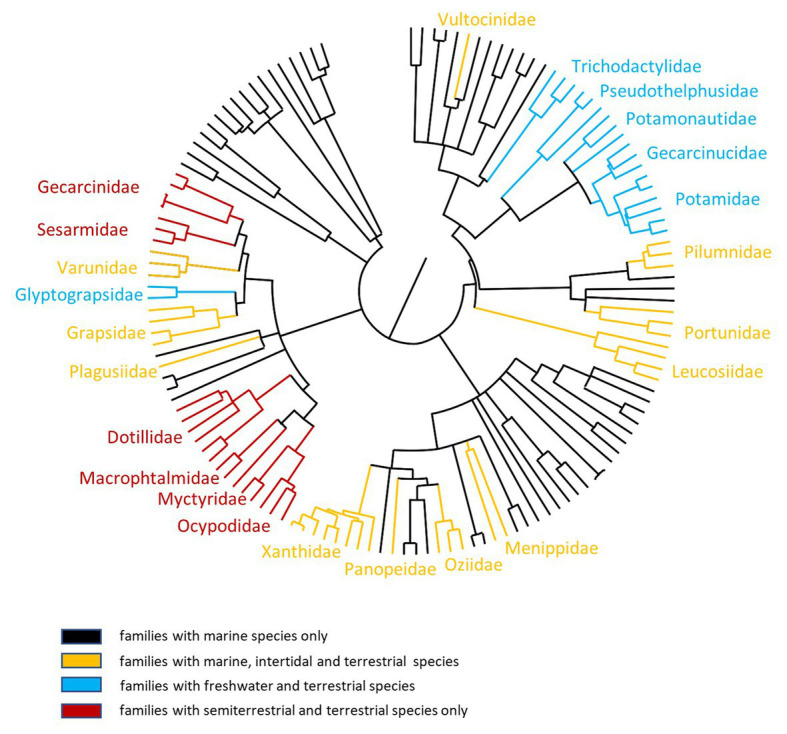
Phylogenetic relationships among semi-terrestrial and terrestrial brachyuran crabs. Unrooted phylogenetic tree of 134 true crab species representative of 57 out of 98 Brachyuran families (see Ng et al., 2008). The order of tree branches is derived from the TimeTree database (http://timetree.org/; Kumar et al., 2017) on the basis of data of Tsang et al. (2014). The color code of the different brachyuran families represents the different habitat they colonized (black lines = families including only marine species; orangelines = families including marine, intertidal and terrestrial species; turquoiselines = families including freshwater and terrestrial species; redlines = families including exclusively semiterrestrial and terrestrial species). Only the taxonomic names of families with terrestrial species are shown on the tree.

The present day high diversity of terrestrial species is the result of a repeated series of independent attempts to conquer terrestrial environments accomplished by several formerly aquatic animal phyla (Little, 1990, 2009; Randall et al., 2009; Lozano-Fernandez et al., 2016). Among all phyla, Arthropoda contributes the largest portion to terrestrial biodiversity. In this group, the conquest of land happened multiple times and the first successful attempts date back to a period in between Cambrian and Silurian (Lozano-Fernandez et al., 2016). Despite its long history, terrestrialization by some groups of arthropods is still an on-going process (Lozano-Fernandez et al., 2016) and in brachyuran crabs is happening right now (Burggren and McMahon, 1988) through a number of independent lineages (Giomi et al., 2014; Figure 1).

**Figure 2:**
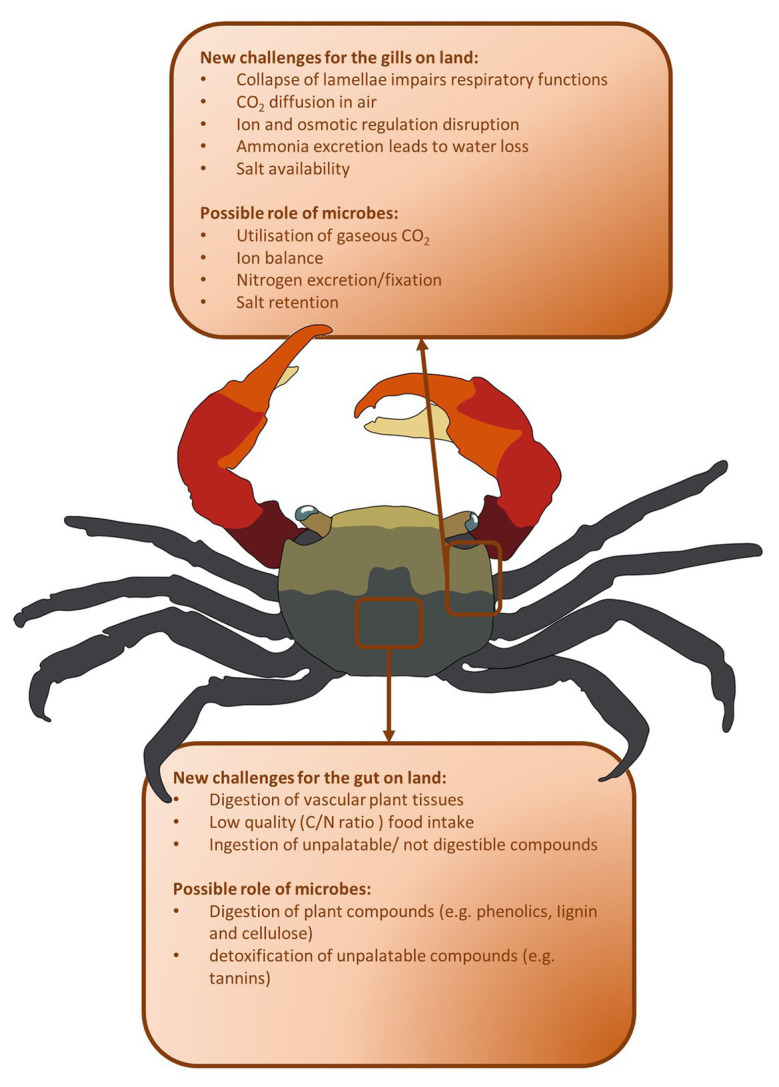
Main challenges met during terrestrialization and possible microbial contribution. Respiratory/urinary and digestive systems are predicted to be the mostly impacted by water-to-land transition. Microbes may help to improve gut functionality toward new nutrient sources (e.g., vascular plant material) and gills functionality in relation to excretion of nitrogen toxic compounds.

The differences between aquatic and terrestrial habitats are enormous and nearly every aspect of a crab life needs to cope with such a transition (Little, 2009). During this process, remarkable morphological and physiological changes were required to tackle challenges relevant to locomotion (Burggren and McMahon, 1988), gaseous exchange (Farrelly and Greenaway, 1993, 1994; Paoli et al., 2015), excretion (Wood and Boutilier, 1985; Greenaway, 1988), reproduction (Cannicci et al., 2011; Simoni et al., 2013), foraging (Lindquist et al., 2009), and salt availability (Anger, 1995; Faria et al., 2017), given the huge difference in physical properties of air and water (Little, 1990; Figure 2).

One of the most remarkable changes that occurred was related to the gills (Figure 2). In marine species, gills perform several functions, such as respiratory activities, ionic and osmotic regulation, pH regulation and, in part, nitrogenous waste excretion. Gills of many intertidal and terrestrial brachyurans lost their respiratory function (Burggren and McMahon, 1988; Little, 1990; Farrelly and Greenaway, 1994) and new respiratory organs were evolved, such as branchiostegal lungs (Farrelly and Greenaway, 2005; Paoli et al., 2015) or tympana on the legs (Maitland, 1986; Figure 2). Once the respiratory role was lost, or greatly reduced, the main physiological functions exerted by the gills are related to ion exchanges, CO_2_ and nitrogen excretion (Morris, 2001; Weihrauch et al., 2004).

Most of terrestrial and semi-terrestrial crabs are mainly relying on vascular plant tissues for food (Linton and Greenaway, 2007) and this new diet is yet another challenge, since their marine ancestors were mainly feeding on micro‐ and macroalgae and on animal preys (Figure 2). Vascular plants have evolved adaptations to prevent herbivory, from deterrence of ingestion, to low digestibility and unpalatability (Wolcott and O’Connor, 1992; Linton and Greenaway, 2007) and are characterized by a low nitrogen/carbon ratio (Linton and Greenaway, 2007). As a consequence, herbivorous terrestrial crabs developed several morphological and physiological adaptations to cope with low nitrogen intake (Linton and Greenaway, 2007; Kawaida et al., 2019; Figure 2). The role of microbes was suggested in support for a low-quality diet, but scarce experimental evidences was gathered (Linton and Greenaway, 2007; Figure 2).

Intertidal brachyuran crabs belonging to unrelated taxa share very similar sensory, respiratory, excretory, and osmoregulatory adaptations to the terrestrial environment (Burggren and McMahon, 1988), indicating convergent evolutionary trends (Little, 2009; Cannicci et al., 2011; Giomi et al., 2014). Microbes are known to be strongly involved in various physiological processes of animals, such as herbivory (see for instance Hansen and Moran, 2014) and ammonia detoxification (van Kessel et al., 2016). However, the role of microbes in the water-to-land evolutionary transition of crabs must still be investigated.

## The Microbial Perspective: The Holobiont Theory and the Evolution of Arthropoda

No multicellular organism is defined by its genes only, but it relies on the genetic functions provided by the microorganisms that are associated to it. This idea has been conceptualized in the holobiont theory of evolution that addresses the host-microbe interaction in an evolutionary perspective. According to this theory, Darwinian evolution acts on the genes of both the host and the commensal microbes (Zilber-Rosenberg and Rosenberg, 2008). Thus, multicellular organisms should be considered as a unique genetic system called hologenome (Zilber-Rosenberg and Rosenberg, 2008; Bordenstein and Theis, 2015) composed by the genetic and functional asset of microorganisms and their host.

Arthropoda represents one of the most relevant animal phyla where host-microbe association has played, and is still playing, a crucial role in environmental adaptation and evolution. In *Drosophila* spp., for instance, variations in the composition of the gut microbiome led to changes in population dynamics that produced allelic divergences, ultimately influencing their ability to adapt to different environments (Rudman et al., 2019). In this phylum, symbiosis can even create paradoxical ecological balances where the benefits of both sides are acquired after a “loss,” such as the emblematic case of *Buchnera aphidicola* that lost entire gene clusters to be better tolerated by the aphid (Houk and Griffiths, 1980), which provides it with essential amino acids. On its side, the aphid gave up some of the genes that help to fight bacterial infections, in order to host this beneficial symbiont. In return, the genome of *B. aphidicola* is tiny, 640.681 bp-640 genes (Shigenobu et al., 2000).

Diet is one of the main factors shaping gut microbiome and is central in the development of stable or transient interactions, critical for the adaptation to the environment. Termites are one of the major examples of organisms depending on resident and cultivated microbiomes (Brune and Dietrich, 2015). They assimilate nutrients from wood substrates (Abe et al., 2000) and play an essential role in ecosystems carbon turnover. This process is made possible by intestinal symbionts which degrade cellulose, hydrolyze xylans and provide CO_2_-reductive acetogenesis and N_2_ fixation (Warnecke et al., 2007).

Ants, more than any other arthropod, are associated to a broad environmental spectrum. Among them, the genera *Acromyrmex* and *Atta* can cultivate symbiotic fungi of the genus *Attamyces* that grow on leaf litter. In this symbiosis, the fungi produce the enzyme laccase (LgLcc1) that allows leaf cutting ants to detoxify phenolic compounds produced by plants (De Fine Licht et al., 2013). In these ant genera, the ant-fungal association is so intimate that it induced a remodeling of the ant genome, which lost arginine biosynthesis genes. On the other hand, fungi positively selected the pathways of chitinase and lost the key domain of ligninase (Nygaard et al., 2016). Furthermore, *Candidatus Westeberhardia cardiocondylae*, symbiont of the ant *Cardiocondyla obscurior*, offers an excellent example of metabolic complementation. The queens retain this microorganism in the ovarian nurse cells and transmit it to the oocytes. As for *B. aphidicola*, the genome of this microorganism also appears drastically reduced (533 kb), and it is responsable for the development of important metabolic complementations. In fact, the symbiont’s ability of producing 4-hydroxyphenylpyruvate, convertible by the host into tyrosine, could contribute to the formation of cuticles during the ant pupal phase (Klein et al., 2016).

Within crustaceans, Isopoda are the most successful group in terms of land colonization and bacteria have been shown to be critical to develop a diet based on vascular plant matrices (Zimmer and Topp, 1998a,b; Zimmer, 2002; Zimmer et al., 2002). Indeed, the ability of several microbial species found in their hepatopancreas to produce Carbohydrate-Active enZymes (CAZymes), involved in lignocellulose degradation is essential for isopods’ adaptation to terrestrial life (Bredon et al., 2019). Specialized microbiomes are also critical for the peculiar diet, based solely on lignocellulose, of the woodboring intertidal isopods *Limnoria* spp. (Besser et al., 2018). The substantial absence of intestinal bacteria in true marine isopods (Zimmer et al., 2001) reinforced the hypothesis that hepatopancreatic symbionts were acquired from the environment during terrestrialization and played a central role in this evolutionary process. Also intertidal amphipods showed to depend on environmentally originated microbiomes for cellulose utilization and species of sand hoppers with different food preferences showed contrasting patterns of cellulose degradation gene abundance in their gut microbiome under controlled feeding conditions (Abdelrhman et al., 2017).

Finally, Cuellar-Gempeler and Leibold (2018, 2019) studied the contribution of surface and burrow sediment bacteria to microbial communities associated with fiddler crabs and found that bacterial communities from burrow sediment colonized the crab carapace, while gut bacterial communities mirrored burrow and surface sediment bacteria. Thus, it has been shown that these intertidal crab species can regulate the bacterial assembly of their gut, but nothing is known about the microbiomes present in the gut of truly terrestrial crabs.

## The Microbial Perspective: Experimental Challenges

Is there a microbial contribution to crab terrestrialization? Did the same microbial taxonomic groups take part in the evolutionary-independent terrestrialization of crabs, or was the provided microbiome functionality (i.e., the ecosystem service due to the microbial part of the holobiont) the main driver (Doolittle and Booth, 2016), irrespective of the taxonomic group? Trying to answer these questions is not trivial and requires defining experimental models and testing the holobiont theory (Moran and Sloan, 2015).

Technically speaking, in the last two decades metagenomics has emerged as a standalone discipline in studying and understanding the functions of microbiomes, showcasing different methods to survey microbial communities in different environment and in association with different hosts. Despite slight differences across sequencing techniques, metagenomic sequencing approaches are divided into two broad categories: marker gene analysis (also called targeted metagenomics) and whole metagenomics sequencing (also called untargeted metagenomics; Knight et al., 2018). The marker gene analysis is quick and cheap and can rely on very large datasets for comparative studies. Typically, it is based on 16S rRNA gene for prokaryotes and Internal Transcribed Spacer (ITS) for fungi. However, taxonomic information is generally provided only at genus level and biases due to PCR amplification can be present. On the contrary, potentially deep (down to the strain level) taxonomic assignment and functional gene profiles (even entire genome sequences) can be disclosed by the untargeted metagenomics approach, but, at present, at higher operational and computational costs than the targeted metagenomics. To gain a real-time view of the functions provided by the microbiome, metatranscriptomic analyses (as a metagenomic representation of genes expressed at each time by the community) have also been exploited (Shakya et al., 2019).

However, despite the recent advances in metagenomics, issues in statistical and experimental design still represent one of the major obstacles when we need to address questions regarding the co-evolution between the host and its microbiome. The two main types of studies commonly used in microbiome researches are cross-sectional studies and longitudinal studies. The first type consists in grouping individuals together based on one or more factors of interest and sampling only at a single specific time-point, making a snapshot of the bacterial community in different individuals. Conversely in the longitudinal studies one or more single individuals are repeatedly sampled over a specific interval of time, giving a dynamic picture of the changes in their microbiome composition during time. Regardless of the type of experiment, a well-defined design and sound biological models are crucial to extract relevant biological information. The development status of the crab, diet, sex, environment, and other crab-specific factors must be accounted to control for variation between groups and to minimize biases due to unbalanced groups (Ramette, 2007). Controlling for natural source of variability as well as technical variability in host-microbiome studies involving animal models is not trivial but a well-defined design can help researchers to minimize biases and disentangle the interaction between the microbiome and its host.

## The Crab Perspective: Comparative and Functional Genomics

Reference genomes proved to be a powerful tool to investigate many biological aspects of target species. In all metazoan taxa, the availability of high-quality reference genomes has led to great advancements in comparative and functional genomics and uncovered several aspects of evolutionary processes of physiological adaptations (Mardis, 2011). The investigation of the terrestrialization process of various groups of organisms also gathered support from genomic data.

Most of the accurate investigation of genomic signature of terrestrialization has been conducted on vertebrates. The analysis of gene expansion and positive selection in the genome of four mudskippers, teleosts uniquely adapted to live on intertidal mudflats, revealed an expansion of innate immune system genes involved in the defense against terrestrial pathogens (You et al., 2014). A positive selection for genes belonging to the ammonia excretion pathway was also detected in the gills, suggesting an important role in mudskippers’ tolerance to both environmental and self-produced ammonia, especially when exposed to air (You et al., 2014). Finally, the loss or mutation of vision-related genes showed genomic changes associated with aerial vision, a pivotal characteristic for terrestrial species (You et al., 2014).

Another extensive comparative genomic analysis conducted on five species of coelacanth fish was able to elucidate the time and mode of the evolutionary trajectories, occurring at molecular level, that lead the transition from fish to tetrapods (Nikaido et al., 2013). This study revealed the presence, in the coelacanth genome, of genes related to the olfactory reception of airborne ligands, unknown in ray-finned fish, and of noncoding elements that act as enhancers of key genes for limb development typical of tetrapods but not of ray-finned fishes (Nikaido et al., 2013).

Investigation of genomic traits involved in the terrestrialization process are scarce in invertebrates. Genomic data produced for four species of Ampullaridae, a family of gastropods that includes both aquatic and amphibious snails, revealed the presence of expanded gene families related to environmental sensing and cellulose digestion, which may have played a key role in the water to land transition in this lineage (Sun et al., 2019).

The above examples show how selective pressures exerted by water-land transition can guide the expansion and/or contraction of specific adaptive genes in the genome of metazoans. Horizontal gene transfer (i.e., the acquisition of new genes from foreign sources) could also lead to genome diversification in the context of terrestrialization. Exchange of genetic materials is commonly reported in bacteria and archaea (Gogarten and Townsend, 2005). The frequency and importance of horizontal gene transfers between bacteria and eukaryotes, however, remained controversial and unclear until recently (Husnik and McCutcheon, 2018). Nonetheless, in the last decade some examples of horizontal gene transfers between bacteria and animals have been proved, showing how this process can be involved in the fixation of functional genes especially linked to the evolution of nutritional requests (see Husnik and McCutcheon, 2018 for a review). For example, horizontal gene transfers of bacterial genes involved in carbohydrate metabolism has been found in herbivorous insects (Wybouw et al., 2016) and plant-parasitic nematodes (Danchin et al., 2010; Paganini et al., 2012). Recently, it has been suggested that the marine wood-boring isopod *Limnoria quadripunctata* and the amphipod *Chelura terebrans* have sterile microbe-free digestive systems and they are able to produce all required enzymes for lignocellulose digestion, showing to possess enzymes previously thought to be absent from animal genomes (King et al., 2010; Kern et al., 2013). These enzymes were likely acquired by these species *via* horizontal gene transfer from a protist symbiont.

## Conclusions and Perspectives

In this review paper, we are promoting intertidal and terrestrial crabs as novel model systems for the understanding of evolutionary mechanisms at the holobiont level. In the context of terrestrialization of crabs, the presence of multiple taxa which independently underwent, and still undergo, the evolutionary leap from sea to land (Figure 1) represents an ideal experimental dataset for cross-sectional studies that aim to compare microbiome composition both among different terrestrial taxa and between them and closely related marine species. Since terrestrialization lead to several, but similar, physiological adaptations (Little, 2009; Cannicci et al., 2011; Giomi et al., 2014), terrestrial crabs represent a suitable model for testing the relationships between microbiome composition and their functions, in order to interpret the ecosystem services provided by the crab-associated microbiome with respect to the new physiological challenges. In terms of organs, the hepatopancreas, where nutrients are stored (Zimmer, 2002), the multifunctional gills, responsible for ion, gas, and nitrogen exchanges (Morris, 2001) and the intestine, which has to cope with non-digestible compounds (Linton and Greenaway, 2007) should be targeted in future research aimed to ascertain the development of host-microbe interactions in these model systems.

The evolution of terrestrialization did not just involve the interaction of crabs with their microbiota, but, necessarily, a selection of specific genomic traits of the crabs themselves. This process is a necessary evolutionary pathway to select for those adaptive traits that play a crucial role in such a dramatic ecological shift. It is also conceivable that genome diversification in crabs that conquered the land could be led by events of horizontal gene transfers. Such transfer events from microbial donors could be likely, for instance, for genes encoding proteins involved in lignin and cellulose degradation, which represents a big challenge in a diet based on vascular plants, as shown for some herbivorous insects (Wybouw et al., 2016).

Conversely to what happened to insects and isopods (Lozano-Fernandez et al., 2016), many lineages of brachyuran crabs are exploiting the evolutionary opportunity of a transition from sea to the land just now (Giomi et al., 2014; Fusi et al., 2016). This transition is happening so rapidly and involves such a diverse array of taxa and habitats, from tree canopies to deserts, that it is difficult to explain such an adaptive radiation without an intimate relationship, at molecular level, between the true crabs and their microbiome.

The use of a multi-disciplinary approach (combining physiology, microbiology, biochemistry) coupled with both targeted and untargeted metagenomics (including metatranscriptomics) can ultimately clarify the contribution of microbiome on crabs terrestrialization and test part of the holobiont theory of evolution, further delving in the heart of darkness of modern evolutionary theory.

## Author Contributions

SC and DC ideated the review. All the authors wrote the manuscript. All authors contributed to the article and approved the submitted version.

### Conflict of Interest

The authors declare that the research was conducted in the absence of any commercial or financial relationships that could be construed as a potential conflict of interest.
